# Performance of Cementless Hip Arthroplasty Stem Types Based on Consolidated Large Registry Data

**DOI:** 10.1016/j.artd.2024.101582

**Published:** 2024-12-14

**Authors:** Logan E. Finger, Matthew F. Gong, Asher Mirvish, Alexandra S. Gabrielli, Ahmad P. Tafti, Michael J. O’Malley, Brian A. Klatt, Johannes F. Plate

**Affiliations:** a Department of Orthopaedic Surgery, University of Pittsburgh, Pittsburgh, PA, USA; b School of Medicine, University of Pittsburgh, Pittsburgh, PA, USA; c School of Health and Rehabilitation Sciences, University of Pittsburgh, Pittsburgh, PA, USA

**Keywords:** Total hip arthroplasty, Stem geometry, Cementless stem, Uncemented stem, Classification system

## Abstract

**Background:**

Improvements in cementless total hip arthroplasty have been directed at optimizing osseointegration of the femoral implant to reduce aseptic loosening rates. Stem design plays a critical role in the performance of these implants. Given the increase in new stem designs and the creation of an updated classification system, improved understanding of the outcomes of each stem type is warranted. The purpose of this study was to determine overall revision rates based on stem design and proprietary model.

**Methods:**

Joint registry data on the reported overall cases and revisions for each cementless stem brand were collected from the annual reports of the American Joint Replacement Registry (2021), United Kingdom National Joint Registry (2021), New Zealand Joint Registry (2020), and Australian Orthopaedic Association National Joint Replacement Registry (2021). Each individual stem brand was classified into a stem type derived from the classification system described by Radaelli et al.

**Results:**

The most utilized stem types were (1) type B2 stems, (2) type A stems, and (3) type C1 stems. The most utilized stem models were the (1) Corail stem (B2), (2) Accolade II (type A), and (3) Taperloc 133 (type A). The highest and lowest overall revision rates observed were in the type B1 stems (8.09%) and type C3 stems (1.12%), respectively. The 3 stem models with the highest overall revision rates were the Synergy HA stem (9.04%), CBC stem (8.59%), and CLS stem (7.96%). The 3 stems with the lowest respective overall revision rates were the C2 stem (0.00%, 0 of 933 cases), Actis Duofix (0.59%), and VerSys stem (0.89%).

**Conclusions:**

Based on consolidated large registry data, some cementless femoral stem types and models appear to perform better than others when compared on the basis of stem design.

## Introduction

**Figure 1:**
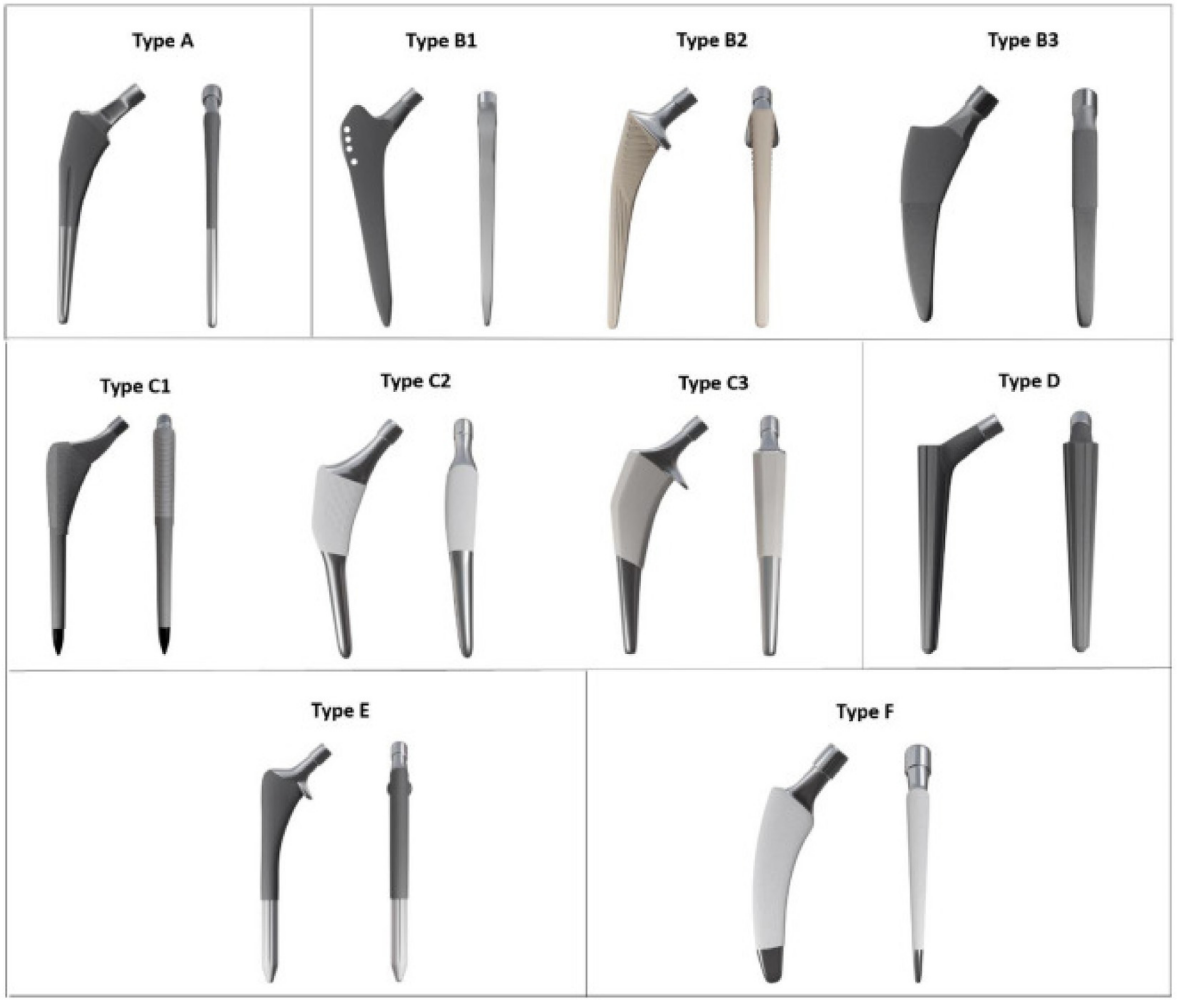
The 6 key stem geometries displayed on the AP and ML planes: flat taper (type A); quadrangular tapers (types B1-B2-B3); fit-and-fill (types C1-C2-C3); conical (type D); cylindrical (type E); and calcar-guided short stems (type F). AP, anteroposterior; ML, mediolateral. From Radealli et al [8].

Popularization of cementless femoral stem fixation has resulted in innovation in stem design. Innovations have been directed toward several specific areas, including (1) improving initial stability at time of initial operation, (2) improving capacity for stem osseointegration over time, and (3) reducing stress shielding and wear induced bone loss to improve implant longevity [[1], [2], [3], [4], [5], [6]]. Several classification systems have been devised in order to capture the wide diversity of stem types which exist on the market [5,7,8]. The classification scheme developed in 2011 by Khanuja et al was the preeminent system initially utilized to classify cementless stem types [5]. More recently, a 2023 update to this previous classification was devised to incorporate some of the more modern stem designs which have recently been developed (Fig. 1) [8]. While this update provided insight and rationale for why the new classification scheme was devised, limited data were provided on usage and revision rate of each of the stem types presented [8]. Understanding the usage and outcomes associated with each specific stem type may assist arthroplasty surgeons to make appropriate stem selections for specific patient anatomy and stem revision rates. Furthermore, reporting stem usage and outcomes data will help surgeons better interpret the 2023 Radaelli classification system, which was designed to supplant the prior Khanuja classification system and incorporates new stem designs developed after 2011. The purpose of this study was to query consolidated national joint registry data to quantify performance of stem types based on a newly developed cementless stem classification scheme. In particular, this study sought to (1) consolidate large national joint registry data for cementless hip stem components and determine overall revision rates based on stem design and (2) to evaluate each of the proprietary implants currently reported in registry data and categorize them appropriately into this novel classification scheme.

## Material and methods

This study was exempt from institutional review board approval. Recent annual reports of several large joint registries were queried to review a consolidated set of data on cementless stem brands. In total, the American Joint Replacement Registry (2021), United Kingdom National Joint Registry (2021), New Zealand Joint Registry (2021), and Australian Orthopaedic Association National Joint Replacement Registry (2021) were all queried [[9], [10], [11], [12]]. Within each joint registry, the section on outcomes of cementless femoral stems was identified, and the number of revisions were recorded by stem model. Stem models were reported based on their usage in primary total hip arthroplasty cases. However, the underlying diagnosis requiring total hip replacement, including osteoarthritis, femoral head osteonecrosis, or hip dysplasia, was not specified in any registry reports. In some registries the combination of stem and acetabular components were reported rather than individual stems. In these registries, the number of cases for each stem regardless of the accompanying acetabular component were combined to simplify the data analysis. All cementless stem brands reported within each joint registry were recorded regardless of volume of usage. If stems from an individual stem brand were reported separately based on presence of HA coating, these results were combined and included together when each stem brand was classified into the scheme presented by Radaelli et al (Fig. 1) [8]. The volume of cases and volume of all-cause revisions were recorded for each stem type. Additionally, the number of all-cause revisions and average follow-up time for each individual stem were calculated from combined registry data. A joint arthroplasty fellowship-trained surgeon then classified each individual proprietary stem based on the Radaelli classification system [8]. Accessible technique guides for each femoral stem were accessed to appropriately classify each stem into a specific type based on the classification system. Total number of cases and revisions were then combined for all stems within a specific stem type, in order to report a consolidated revision rate. In all analyses, revision rate reported as a percentage by assessing the number of revisions reported as a proportion of the total number of stems originally implanted.

### Data analyses

To ensure that an adequate number of cases for each stem model were included such that a few poor outcomes would not skew the failure rates, an arbitrary 500 case minimum was imposed to be included in the data analysis. The models that were excluded are listed and underlined in Table 1. Revision rates were compared using a *chi*-square test to determine if there was any statistically significant difference between the stem type categories or stem models. Additionally, *post hoc* pairwise *Z*-tests of proportions were performed to assess differences in revisions rates between stem types and models. This analysis was performed using a Bonferroni correction with alpha = 0.05. Any continuous variables were analyzed using a 1-way analysis of variance test with post-hoc Tukey honestly significant difference, where applicable. All analyses were performed using IBM SPSS.

**Table 1:** List of proprietary stem models included in the national registries as well as their stem type, manufacturer, usage total and proportion and revision total and rate.

Type	Stem model	Stem manufacturer	Total	Revision total	Revision rate (%)	Average follow-up (y)
A	ABG II	Stryker, Kalamazoo, MI	1965	139	7.1	9.70
A	ABG	Stryker, Kalamazoo, MI	188	60	31.9	16.75
A	Accolade II	Stryker, Kalamazoo, MI	71,179	1356	1.9	5.02
A	Accolade TMZF	Stryker, Kalamazoo, MI	3091	65	2.1	7.00
A	Accolade	Stryker, Kalamazoo, MI	29,471	1657	5.6	14.55
A	Anthology	Smith & Nephew, Memphis, TN	24,401	708	2.9	8.85
A	Linear	DJO Global Inc., Lewisville, TX	2157	20	0.9	7.00
A	M/L Taper Kinectiv	Zimmer Biomet, Warsaw, IN	938	24	2.6	7.00
A	M/L Taper	Zimmer Biomet, Warsaw, IN	34,119	886	2.6	7.64
A	MasterSL	LimaCorporate, San Daniele, Italy	117	3	2.6	1.99
A	Ovation Hip Stem	ODEV, Draper, UT	848	8	0.9	5.00
A	TaperFill	DJO Global Inc., Lewisville, TX	972	12	1.2	5.00
A	Taperloc 133	Zimmer Biomet, Warsaw, IN	51,133	1049	2.1	5.65
A	Taperloc Microplasty	Zimmer Biomet, Warsaw, IN	9640	138	1.4	5.50
A	Tri-Fit TS	Corin Group, Cirencester, England, UK	4150	87	2.1	8.83
A	Tri-Lock	DePuy Synthes, Raynham, MA	15,888	229	1.4	7.62
B1	Alloclassic	Zimmer Biomet, Warsaw, IN	6661	303	4.5	4.33
B1	C2	LimaCorporate, San Daniele, Italy	933	0	0.0	7.50
B1	CBC	Mathys Medical, Bettlach, Switzerland	687	59	8.6	8.93
B1	CLS	Zimmer Biomet, Warsaw, IN	10,352	824	8.0	13.19
B1	SL-Plus	Smith & Nephew, London, England, UK	6803	494	7.3	5.00
B2	Alteon	Exactech, Gainesville, FL	803	17	2.1	3.00
B2	Avenir-Muller	Zimmer Biomet, Warsaw, IN	5630	140	2.5	7.93
B2	Corail	DePuy Synthes, Raynham, MA	318,729	18,520	5.8	9.38
B2	EVOK	Amplitude Ortho, Valence, France	1459	39	2.7	5.00
B2	H-Max M (Modular)	LimaCorporate, San Daniele, Italy	157	12	7.6	9.14
B2	H-Max S	LimaCorporate, San Daniele, Italy	2837	109	3.8	4.82
B2	HACTIV	Evolutis S.A.S, Briennon, France	1609	45	3.8	4.00
B2	Metafix	Corin Group, Cirencester, England, UK	17,734	364	2.1	5.39
B2	Origin	DJO Global Inc., Lewisville, TX	1959	50	2.6	15.00
B2	Paragon	Corin Group, Cirencester, England, UK	5495	102	1.9	5.00
B2	PolarStem (Uncemented)	Smith & Nephew, Memphis, TN	40,467	804	2.0	6.70
B2	Profemur L	MicroPort Orthopedics, Arlington, TN	2713	103	3.8	5.00
B2	Quadra-H (HA Coated)	Medacta International, Castel San Pietro, Switzerland	15,179	505	3.3	3.55
B2	Stemsys (Uncemented)	Evolutis, Briennon, France	3055	70	2.3	4.73
B2	Twinsys (Uncemented)	Mathys Medical, Bettlach, Switzerland	8585	459	5.3	6.47
B3	Fitmore	Zimmer Biomet, Warsaw, IN	1880	48	2.6	7.00
C1	Citation	Stryker, Kalamazoo, MI	1177	45	3.8	7.00
C1	Echo Bi-Metric	Zimmer Biomet, Warsaw, IN	4432	87	2.0	4.77
C1	Furlong HAC	JRI Orthopedics, Sheffield, England, UK	41,649	1648	4.0	13.5
C1	Mallory Head	Zimmer Biomet, Warsaw, IN	105	17	16.2	13.23
C1	Novation	Exactech, Gainesville, FL	1306	16	1.2	7.00
C1	Omnifit (Uncemented)	Rontis Medical, Zug, Switzerland	1272	99	7.8	10.00
C1	Secur-Fit Max	Stryker, Kalamazoo, MI	3328	77	2.3	7.00
C1	Secur-Fit	Stryker, Kalamazoo, MI	13,132	555	4.2	10.00
C1	Secure-Fit Plus Max	Stryker, Kalamazoo, MI	8859	298	3.4	13.50
C1	Summit	DePuy Synthes, Raynham, MA	31,275	748	2.4	9.77
C1	Synergy HA	Smith & Nephew, Memphis, TN	2257	204	9.0	7.00
C1	Synergy	Smith & Nephew, Memphis, TN	26,132	846	3.2	9.43
C1	Trabecular Metal	Zimmer Biomet, Warsaw, IN	4158	102	2.5	7.04
C3	Actis DuoFix	DePuy Synthes, Raynham, MA	9385	55	0.6	3.00
C3	AMIStem-H	Medacta International, Castel San Pietro, Switzerland	5914	117	2.0	5.50
D	SL Monoblock	Zimmer Biomet, Warsaw, IN	488	27	5.5	11.64
D	Wagner Cone Stem	Zimmer Biomet, Warsaw, IN	133	6	4.5	15.65
E	AML	DePuy Synthes, Raynham, MA	53	9	17.0	15.80
E	Prodigy	DePuy Synthes, Raynham, MA	113	25	22.1	13.82
E	VerSys	Zimmer Biomet, Warsaw, IN	7506	67	0.9	11.00
F	Furlong Evolution (Cementless)	JRI Orthopedics, Sheffield, England, UK	5133	109	2.1	5.00
F	MiniHip	Corin Group, Cirencester, England, UK	3594	126	3.5	3.00
F	Nanos	Smith & Nephew, Memphis, TN	660	10	1.5	7.50
F	Optimys	Mathys Medical, Bettlach, Switzerland	1805	26	1.4	3.43
Mod	Apex (Modular)	Apex Orthopedics & Sports Medicine, Overland Park, KS	1529	81	5.3	15.00
Mod	S-ROM (Modular)	DePuy Synthes, Raynham, MA	7781	435	5.6	10.98
Mod	SL Modular	Zimmer Biomet, Warsaw, IN	405	45	11.1	14.87

Underlining indicates exclusion from data analysis due to lack of sufficient case numbers.

Mod, modular.

## Results

In total, 65 cementless stem models (Table 1) were included based on our query of registry data. Reported usage, follow-up duration and revision rates were collected from each registry and consolidated.

### Usage rates and follow-up data

Inclusive of the stems with fewer than 500 cases recorded overall, the type B2 and type A stems are most represented by existing proprietary stem models, with 16 each. The number of these stem models appeared to match utilization rates, as the most utilized stem types were type B2 stems (429,752 cases), type A stems (247,198), and type C1 stems (101,954 cases). The average follow-up duration for each individual stem (Table 1) and stem type category (Table 2) was calculated. No statistically significant difference was found between the average follow-up durations of the stem categories (*P* value .17) (Table 2) In terms of specific stem brands, the most utilized stem brands were Corail (type B2, 318,729 cases), Accolade II (type A, 71,179), and Taperloc 133 (type A, 51,133) (Table 3).

**Table 2:** Average follow-up duration of each stem type excluding stems without at least 500 cases reported cumulatively and statistical significance.

Stem type	Average follow-up duration (years)	Std. Dev.	*P* value
Type A	7.53	2.61	.16
Type B1	9.42	4.60	
Type B2	6.89	3.07	
Type B3	7.00	n/a	
Type C1	7.92	2.41	
Type C2	n/a	n/a	
Type C3	4.25	1.77	
Type D	n/a	n/a	
Type E	11.00	n/a	
Type F	3.48	0.50	
Modular	7.65	4.70	

n/a, not available; std. dev., standard deviation.

**Table 3:** Number of available stems in each category, usage of each stem type and most used stem brands in each category inclusive of models with fewer than 500 cases.

Type	# Of stems	# Of total cases	Most common models
Type A	16	251,443	Accolade II (99,523) Taperloc 133 (51,133)
Type B1	5	25,436	CLS (10,352) SL-Plus (6803)
Type B2	16	456,923	Corail (348,096) PolarStem (40,856)
Type B3	1	1880	Fitmore (1880)
Type C1	13	139,082	Furlong HAC (41,649) Summit (31,275)
Type C2	0	0	N/A
Type C3	2	15,299	Actis DuoFix (9385) AMIStem-H (5,914)
Type D	2	621	SL Monoblock (488) Wagner Cone Stem (133)
Type E	3	7944	VerSys (7778) Prodigy (113)
Type F	4	11,192	Furlong Evolution (5133) MiniHip (3594)
Modular	3	9715	S-ROM (7781) Apex (1529)

### Revision rates

*Chi*-square analysis revealed a statistically significant difference in revision rates between the different stem types (*P* value < .01) (Table 4). The overall revision rate based on the incidence within the compiled registry data was 4.19%. Amongst consolidated stem types, the highest overall revision rates observed were in type B1 stems (8.09%, 1680 of 20,776 cases), type B2 stems (4.97%, 21,339 of 429,595 cases), and type C1 stems (4.66%, 4742 of 101,849). The lowest overall revision rates were observed in the type C3 stem (1.12%, 172 of 15,299 cases), the type E stem (1.35%, 101 of 7506 cases), and the type B3 stem (2.55%, 48 of 1880 cases). *Post hoc* Z-tests of proportions were performed in order to determine which individual stem types included in the *Chi-*square analysis were statistically different from the others. This analysis identified 6 unique groups of stems with revision rates similar to the other stems in that distinct group: group 1 – type A (2.63%) and B3 (2.55%) stems (*P* value >.05); group 2 – type B1 (8.09%) stems (*P* value >.05); group 3 – type B2 (4.97%) and F (4.12%) stems (*P* value >.05); group 4 – type B3 (2.55%) and F (4.12%) and modular (3.88%) stems (*P* value >.05); group 5 – type C1 (4.66%) and F (4.12%) stems (*P* value >.05); and group 6 – type C3 (1.12%) and E (1.35%) stems (*P* value >.05) (Table 5).

**Table 4:** Absolute number of revisions and revision rate for consolidated stem types exclusive of stems with fewer than 500 total cases as well as statistical significance of difference in rate.

Type	Case total	Revision total	Revision rate (%)	*P* value
Type A	246,893	6483	2.63	<.01^a^
Type B1	20,776	1680	8.09	
Type B2	429,595	21,339	4.97	
Type B3	1880	48	2.55	
Type C1	101,849	4742	4.66	
Type C2	0	0	N/A	
Type C3	15,299	172	1.12	
Type D	0	0	N/A	
Type E	7506	101	1.35	
Type F	6584	271	4.12	
Modular	14,442	561	3.88	

a Statistical significance.

**Table 5:** Groupings of stem types with similar revision rates as determined by *post hoc* Z-test of proportions.

Grouping	Revision rate (%)
Group 1	
Type A	2.63
Type B3	2.55
Group 2	
Type B1	8.09
Group 3	
Type B2	4.97
Type F	4.12
Group 4	
Type B3	2.55
Type F	4.12
Modular	3.88
Group 5	
Type C1	4.66
Type F	4.12
Group 6	
Type C3	1.12
Type E	1.35

Amongst the proprietary stem models, the 3 stems with the highest overall revision rates were the Synergy HA stem (9.04%, 204 of 2257 cases), CBC stem (8.59%, 59 of 687 cases), and CLS stem (7.96%, 824 of 10,352 cases). The 3 stems with the lowest respective overall revision rates were the C2 stem (0.00%, 0 of 933 cases), Actis Duofix (0.59%, 55 of 9385 cases), and VerSys stem (0.89%, 67 of 7506 cases). A c*hi*-square analysis comparing the revision rates of the top 10 used stems revealed a statistically significant difference (*P*-value <.01). Of these 10 specific stems, 3 had a higher-than-expected revision rate. These were the Corail, Furlong HAC, and Accolade stems (Table 6). The Z-tests of proportions also showed that the revision rates of the Corail and Accolade stems were significantly different compared to any other of the 8 stems. All significantly different groups identified are as follows: group 1 – Corail (5.81%) and Accolade (5.62%) (*P* value >.05); group 2 – Accolade II (1.91%), Taperloc 133 (2.05%), and PolarStem (1.99%) (*P* value .05); group 3 – Taperloc 133 (2.05%) and Summit (2.39%) (*P* value >.05); group 4 – Furlong HAC (3.96%) (*P* value >.05); group 5 – M/L Taper (2.60%) and Anthology (2.90%) (*P* value >.05); group 6 – M/L Taper (2.60%) and Summit (2.39%) (*P* value >.05); and group 7 – Synergy (3.24%) and Anthology (2.90%) (*P* value >.05) (Table 7). The average follow-up duration for the aforementioned stem brands was found to be statistically different (*P* value .02) and the *post hoc* Tukey honestly significant difference test revealed that the Accolade stem was the reason for the difference. When the Accolade is excluded as well as the Furlong HAC, since there was only one timepoint for follow up, no difference in follow-up duration was observed.

**Table 6:** Absolute number of revisions and revision rate for the top-10 most used uncemented stems as well as statistical significance of difference in revision rate.

Type	Stem brand	Total cases	Usage rate (%)	Total revisions	Revision rate (%)	*P* value
Type B2	Corail	318,729	32.16	18,520	5.81	<.01^a^
Type A	Accolade II	71,179	7.18	1356	1.91	
Type A	Taperloc 133	51,133	5.16	1049	2.05	
Type C1	Furlong HAC	41,649	4.20	1648	4.96	
Type B2	PolarStem	40,467	4.08	804	1.99	
Type A	M/L Taper	34,119	3.44	886	2.60	
Type C1	Summit	31,275	3.16	748	2.39	
Type A	Accolade	29,471	2.97	1657	5.62	
Type C1	Synergy	26,132	2.64	846	3.24	
Type A	Anthology	24,401	2.46	708	2.90	

a Statistical significance.

**Table 7:** Groupings of stem models with similar revision rates as determined by *post hoc* Z-test of proportions.

Grouping	Revision rate (%)
Group 1	
Corail	5.81
Accolade	5.62
Group 2	
Accolade II	1.91
Taperloc 133	2.05
PolarStem	1.99
Group 3	
Taperloc 133	2.05
Summit	2.39
Group 4	
Furlong HAC	3.96
Group 5	
M/L Taper	2.60
Anthology	2.90
Group 6	
M/L Taper	2.60
Summit	2.39
Group 7	
Synergy	3.24
Anthology	2.90

The rates of the top 3 utilized stem models in each stem category, if there were sufficient stems in each group, were also analyzed to determine if their revision rates varied significantly from each other. The revision rates for individual stem models for all stem types (*P* value <.01) other than modular stems (*P* value .55) were found to be statistically different based on *Chi-*square analysis. Additionally, within the groups found to be statistically different, a *Z-test* of proportions was performed to identify which stem(s) led to the difference in revision rates being statistically significant (*P* value <.05) (Table 8). The revision rates of all the stems that had at least 500 cases reported within a certain stem class were compared and there was no significant difference found between stems (type A: *P* value = 1.0, type B1: *P* value = 1.0, type B2: *P* value = 1.0, type C1: *P* value = 1.0, type C3: *P* value 1.0, type F: *P* value = 1.0). Types B3, C2, D, and E were excluded from this analysis due to either no more than 1 stem being available for analysis or, if there was more than 1 stem available, the number of cases they were used in was less than 500.

**Table 8:** Absolute number of revisions and revision rate for the most utilized stems in each stem category as well as statistical significance of difference in revision rate (no type C2 or D stems were available for comparison due to exclusion criteria).

Type	Total cases	Total revisions	Revision rate (%)	*P* value
Type A				<.01^a^
Accolade II	71,179	1356	1.91	
Taperloc 133	51,133	1049	2.05	
M/L Taper	34,119	886	2.60	
Type B1				<.01^a^
SL-Plus	6803	494	7.27	
Alloclassic	6661	303	4.55	
CLS	10,352	824	7.96	
Type B2				<.01^a^
Corail	318,729	18,520	5.81	
PolarStem	40,467	804	1.99	
Metafix	17,734	364	2.05	
Type C1				<.01^a^
Furlong HAC	41,649	1648	3.96	
Summit	31,275	748	2.39	
Synergy	26,132	846	3.24	
Type C3				<.01^a^
Actis DuoFix	9385	55	0.59	
AMIStem-H	5914	117	1.98	
Type F				<.01^a^
Furlong Evolution	5133	109	2.13	
MiniHip	3594	126	3.50	
Optimys	1805	26	1.44	
Modular				.55
S-ROM	7781	445	5.59	
Apex	1529	81	5.30	

a Statistical significance for *chi-*square evaluation, underlining indicates statistically similar revision rates based on *post hoc* Z-test of proportions with Bonferroni Correction.

## Discussion

Greater understanding of overall usage and outcomes associated with particular stem designs has important implications for primary total hip arthroplasty, as it can help a surgeon appraise which stems are appropriate for each individual patient. Combining data from multiple national registries allows for the creation of a quasi-international database that is more generalizable to many patient populations. Additionally, combining multiple registries increases the sample size for each stem, which thereby decreases the likelihood that a few negative outcomes in a single center or one particular region will skew the outcomes. Furthermore, this strategy allows for stems that are not routinely available in one region to be included and discussed, and depending on the results of such data, may inform a surgeon to seek out a different model that has been shown to perform well.

### Usage rates

Type B2, A and C1 stems were the most utilized stems identified in our study, which is directly correlated with the number of proprietary stems available in each of these categories. Our consolidated registry data identified 16 different B2 and A stems, as well as 13 different C1 stems produced by industry. Of the top 10 most utilized stems, all were type B2, A or C1 stems, which illustrates industry’s focus as well as surgeon preference for stems with quadrangular taper, flat-wedge, or fit-and-fill morphology. The popularity of these types of stems may also suggest a geometrical stem configuration which may be compatible with a wider range of normal patient anatomy. Amongst the proprietary stem models available, the Corail stem, the Accolade II and the Taperloc 133 were the most utilized stem models, with the Corail system having a usage rate over 3 times greater than the Accolade II. Implant longevity is an additional factor critical to a surgeon’s perception of a stem’s reliability, and each of these high-usage stems are associated with low revision rates as detailed in the following section.

### Revision rates

Overall, our consolidated analysis found that the type C3 short fit-and-fill stem (1.12%) and type E cylindrical stem (1.35%) had the lowest revision rates amongst all stem types. Comparatively, the type B1 rectangular taper stem (8.09%) was found to have a statistically higher revision rate among all stem types. The high-usage stems comprising the type B2 quadrangular taper (4.97%), type A flat-wedge (2.63%), and type C1 fit-and-fill (4.66%) stems all had low revision rates consistent with their usage popularity.

The type C3 and type E stems demonstrated the lowest overall revision rates in our study’s findings. Compared with other stem designs, the type C3 stem is a relatively new design with few models currently available on the market. Representative stems, including the Actis DuoFix and AMIStem-H have shown similar clinical, radiographic, and patient-reported outcomes as their longer-stem type C1 and C2 counterparts in prior studies [13,14]. However, studies reported on type C3 stems with medium to long-term follow-up have shown more variable findings. Previous state registry and systematic review studies reported revision rates of the Actis DuoFix (1.08% at 3-year follow-up) and AMIStem-H (1.20% at 5-year follow-up) which are comparable to this study [15,16]. Comparatively, a single-center study of the AMIStem-H noted a relatively high revision rate of 4.2% at 5-year follow-up, most notably due to early aseptic loosening [13]. The most commonly used type E stem, the Versys stem, has reported all-cause revision rates of <1%-2.2% in the literature [17,18]. However, a high rate of complications, particularly intra-operative fracture, has also been reported in this stem and is not necessarily reflected in these relatively low reported revision rates [18]. Nonetheless, prior studies support the low overall revision rates of the type C3 and type E stem as identified in this study. However, studies with longer-term follow-up and an understanding of specific diagnoses in which these stems were indicated for primary total hip arthroplasty are needed to better guide surgeon usage.

The type B1 rectangular taper stem was found to have a higher revision rate (8.09%) compared to all other stems in this study. Amongst proprietary models within this stem category, the Alloclassic stem had the lowest rate of revision (4.55%). A prior 30-year follow-up study noted around 4% of patients with an Alloclassic stem required revision, which is similar to the rate reported in our study [19]. Other type B1 stems such as the SL-Plus and CLS have high reported revision rates in the literature, and may contribute to the higher revision rate of 8.09% [20,21]. A prior comparative study also noted greater stress shielding in the SL-Plus compared to the Alloclassic [20]. Stress shielding-induced bone loss may increase exposure of the bone-stem interface to wear particle-induced loosening, potentially explaining a greater risk of revision due to aseptic loosening. Unfortunately, lack of context in terms of diagnoses and indications for use of this stem in the primary setting make it difficult to appraise when these stems should be used. However, a high rate of complications, particularly intra-operative fracture, has also been reported in this stem and is not necessarily reflected in these relatively low reported revision rates.

As previously noted, the high-usage rate stems comprising of the type B2 (4.97%), A (2.63%), and C1 (4.66%) all had relatively low associated revision rates. As the most highly utilized proprietary stem overall, the Corail (B2) (5.81%) was simultaneously the stem most likely to be revised within the top 10 most-used models. Prior studies detailing the survivorship of the Corail stem corroborate the revision rate of this study of ∼5% [22,23]. However, given this high usage rate, a likely major confounding factor may be the contribution of cases to registry data performed by surgeons with relatively less arthroplasty experience, including fellowship training. Nonetheless, stem design could certainly be contributory to this high reported revision rate. In comparison, the PolarStem, another B2 stem, had a significantly lower revision rate of 1.99% compared to the Corail stem. Prior studies assessing the survivorship of the PolarStem note similar all-cause revision rates of approximately 2% [24,25]. Similarly, although the Accolade II (A) and Taperloc 133 (A) stem are 2nd and 3rd in overall usage, the Corail stem total case volume is approximately 4-6 times the volume of these stems, and more prone to confounding by cases performed by less experienced surgeons. Both the Accolade II and Taperloc 133 stems have low revision rates of 1.91% and 2.05%, respectively, which reflect prior studies evaluating these stems [26,27]. The Furlong hydroxyapatite-coated stem, which is the most utilized C1 stem, also carries a relatively high revision rate of 4.96%, and is above our study’s average compiled registry revision rate of 4.19%. Amongst these stem types with high usage rates, our study found the Accolade II (A), Taperloc 133 (A), and PolarStem (B2) to have relatively low revision rates, and are suitable options for primary total hip arthroplasty unless patient proximal femoral morphology dictates otherwise.

### Limitations

This study has many limitations, particularly those which are inherent to utilizing large, national registry data. Using consolidated registry data can be limited by variable reporting of outcomes by individual registry, limited reporting of patient-specific factors, and lack of information regarding circumstances of stem/implant failure. A notable patient-specific factor was the lack of specific diagnoses associated with use of each particular stem, which would better contextualize how stems more routinely used in the revision setting were implemented in a primary total hip arthroplasty case. Additionally, the availability of particular stem types and surgeon preferences may differ between the countries of each originating joint registry. Therefore, certain stems were not reported in all the national registries queried in this study. Additionally, some of the national registries (eg, AJRR) have a 2500 case minimum for a stem implant to be included in the registry. Other registries include every stem regardless of usage volume. To ensure that an adequate number of cases for each stem model were included such that a few poor outcomes would not skew the failure rates, an arbitrary 500 case minimum was imposed for this study. This study methodology may also predispose to greater contribution to results from registries with higher case volumes such as the AJRR. Each registry also varied in terms of reported follow-up time for each stem, which may skew the revision rates reported. In this study, stems that have been in use longer may have more reported revisions compared to stems newer to the market with less follow-up time. Specific stem and acetabular component combinations, as well as bearing choice, also were not assessed in this study and are a major limitation. This study assessed all-cause revision rates independent of stem-cup combination out of necessity, as distinction between stem and cup failure could not be performed as not all registries report if occurrence of failure was stem- or cup-specific. In summary, although consolidated registry data does not offer a level of granularity needed to answer more specific questions related to stem performance, this study’s focus on overall revision rates relating to stem design is certainly of interest to the greater arthroplasty community. Without question, more granular data is valuable, and this study seeks to lay a foundation for more detailed research which can be pursued exploring the utility of this classification system and understanding stem design’s effect on performance.

## Conclusions

While this study has limited capability to establish complete causality between specific stem types and models and risk for revision due to the nature of using registry data, some cementless femoral stem components appear to perform better than others when compared on the basis of stem design. Additional research is warranted to elucidate if specific stem types are optimized for particular patient populations or femur morphology. With an updated classification scheme, comparing outcomes of different stem types will be more facile moving forward, thereby providing a foundation to better evaluate and compare the performance of different stem designs.

## Conflicts of interest

Frank Johannes Plate reports being a paid consultant for a Smith & Nephew; having stock or stock options in Eventum Orthopedics; receiving research support from Osteal Therapeutics; having a medical/orthopedic publications editorial/governing board membership of the Journal of Arthroplasty; and being a board member/committee appointments for AAHKS research committee. Brian A. Klatt reports receiving other financial or material support from Biomet, Depuy, A Johnson & Johnson Company, Smith & Nephew, Stryker, and Zimmer; receiving royalties, financial or material support from SLACK Incorporated; being a part of medical/orthopedic publications editorial/governing board membership of the Journal of Arthroplasty, Clinical Orthopedics and Related Research, Journal of American Academy of Orthopaedic Surgeons; and being a board member/committee appointments for American Academy of Orthopaedic Surgeons, AAOS/AAHKS Abstract Review Committee, AAHKS, and MSIS. Michael O’Malley reports being a paid consultant for Smith & Nephew and Stryker. Ahmad P. Tafti reports being a board member of IEEE Computer Society and being a speakers bureau for CMU-Pitt Micro-Course. All other authors declare no potential conflicts of interest.

For full disclosure statements refer to https://doi.org/10.1016/j.artd.2024.101582.

## CRediT authorship contribution statement

**Logan E. Finger:** Writing – review & editing, Writing – original draft, Investigation, Formal analysis. **Matthew F. Gong:** Writing – original draft, Formal analysis, Data curation. **Asher Mirvish:** Formal analysis, Data curation. **Alexandra S. Gabrielli:** Writing – original draft, Formal analysis, Data curation. **Ahmad P. Tafti:** Writing – review & editing, Supervision. **Michael J. O’Malley:** Writing – review & editing, Validation, Project administration. **Brian A. Klatt:** Writing – review & editing, Validation, Project administration, Conceptualization. **Johannes F. Plate:** Writing – review & editing, Writing – original draft, Validation, Supervision, Project administration, Conceptualization.

## Appendix A Supplementary data

Conflict of Interest Statement for Plate

Conflict of Interest Statement for Gabrielli

Conflict of Interest Statement for Mirvish

Conflict of Interest Statement for Klatt

Conflict of Interest Statement for Finger

Conflict of Interest Statement for Gong

Conflict of Interest Statement for O’Malley

Conflict of Interest Statement for Tafti
