# Supplementary material for: Performance of Cementless Hip Arthroplasty Stem Types Based on Consolidated Large Registry Data
Source: Arthroplast Today. 2024 Dec 14;31:101582. doi: 10.1016/j.artd.2024.101582 (PMC11715119; doi:10.1016/j.artd.2024.101582)
Supplement: Conflict of Interest Statement for Plate [file mmc1.docx]

# INDIVIDUAL CONFLICT OF INTEREST STATEMENT

***American Association of Hip and Knee Surgeons***

(Adopted from the American Academy of Orthopaedic Surgeons disclosure statement)

The following form **must be filled out completely and submitted by each author (example, 6 authors, 6 forms).**

**All items require a response. If there is no relevant disclosure for a given item, enter "*None*.”**

Performance of Cementless Hip Arthroplasty Stem Components by Type Based on Consolidated Large Registry Data

**Manuscript Title**

1. Royalties from a company or supplier (The following conflicts were disclosed) none

2. Speakers bureau/paid presentations for a company or supplier (The following conflicts were disclosed) none

3A. Paid employee for a company or supplier (The following conflicts were disclosed) none

3B. Paid consultant for a company or supplier (The following conflicts were disclosed) Smith & Nephew

3C. Unpaid consultants for a company or supplier (The following conflicts were disclosed) none

4. Stock or stock options in a company or supplier (The following conflicts were disclosed) Eventum Orthopedics

5. Research support from a company or supplier as a Principal Investigator (The following conflicts were disclosed) Osteal Therapeutics

6. Other financial or material support from a company or supplier (The following conflicts were disclosed) none

7. Royalties, financial or material support from publishers (The following conflicts were disclosed) none

8. Medical/Orthopaedic publications editorial/governing board (The following conflicts were disclosed) Journal of Arthroplasty

9. Board member/committee appointments for a society (The following conflicts were disclosed) AAHKS research committee

**Each author must sign AND print or type his/her name, date and submit a separate form**

In addition, one BLINDED Conflict of Interest form (no author names used) should be submitted per manuscript with all author disclosures.

Frank Johannes Plate Johannes Plate 9/27/24

Author Name (Print or Type) Author Signature Date
