# Supplementary material for: Performance of Cementless Hip Arthroplasty Stem Types Based on Consolidated Large Registry Data
Source: Arthroplast Today. 2024 Dec 14;31:101582. doi: 10.1016/j.artd.2024.101582 (PMC11715119; doi:10.1016/j.artd.2024.101582)
Supplement: Conflict of Interest Statement for Klatt [file mmc4.pdf]

INDIVIDUAL CONFLICT OF INTEREST STATEMENT

American Association of Hip and Knee Surgeons

(Adopted from the American Academy of Orthopaedic Surgeons disclosure statement)

The following form **must be filled out completely and submitted by each author (example, 6 authors, 6 forms). All items require a response. If there is no relevant disclosure for a given item, enter "*None*."**

Performance of Cementless Hip Arthroplasty Stem Components by Type Based on Consolidated Large Registry Data

Manuscript Title

1. Royalties from a company or supplier (The following conflicts were disclosed)

None.

2. Speakers bureau/paid presentations for a company or supplier (The following conflicts were disclosed)

None.

3A. Paid employee for a company or supplier (The following conflicts were disclosed)

None.

3B. Paid consultant for a company or supplier (The following conflicts were disclosed)

None.

3C. Unpaid consultants for a company or supplier (The following conflicts were disclosed)

None.

4. Stock or stock options in a company or supplier (The following conflicts were disclosed)

None.

5. Research support from a company or supplier as a Principal Investigator (The following conflicts were disclosed)

None.

6. Other financial or material support from a company or supplier (The following conflicts were disclosed)

Biomet – Other financial or material support.
Depuy, A Johnson & Johnson Company – Other financial or material support.
Smith & Nephew – Other financial or material support.
Stryker – Other financial or material support.
Zimmer – Other financial or material support.

7. Royalties, financial or material support from publishers (The following conflicts were disclosed)

SLACK Incorporated – Publishing royalties, financial or material support.

8. Medical/Orthopaedic publications editorial/governing board (The following conflicts were disclosed)

Clinical Orthopaedics and Related Research – Editorial or governing board.
Journal of Arthroplasty – Editorial or governing board.
Journal of the American Academy of Orthopaedic Surgeons: Editorial or governing board.

9. Board member/committee appointments for a society (The following conflicts were disclosed)

AAOS – Board or committee member
AAOS/AAHKS Abstract Review Committee – Board or committee member
American Association of Hip and Knee Surgeons – Board or committee member
MSIS – Board or committee member

Each author must sign AND print or type his/her name, date and submit a separate form

In addition, one BLINDED Conflict of Interest form (no author names used) should be submitted per manuscript with all author disclosures.

Brian A. Klatt	Brian Klatt	9/27/24
Author Name (Print or Type)	Author Signature	Date
